# To Increase Mental Health Literacy and Human Rights Among New-Coming, Low-Educated Mothers With Experience of War: A Culturally, Tailor-Made Group Health Promotion Intervention With Participatory Methodology Addressing Indirectly the Children

**DOI:** 10.3389/fpsyt.2020.00611

**Published:** 2020-07-08

**Authors:** Solvig Ekblad

**Affiliations:** Department of Learning, Informatics, Management and Ethics, Cultural Medicine, Karolinska Institutet, Stockholm, Sweden

**Keywords:** trauma, literacy, refugee, women—health and hygiene, low-education households, intervention and evaluation

## Abstract

Due to the increasing numbers of newcomers with impacts of war, civil war and persecution, at high risk of trauma-related mental health problems, there is a need for increase the poor mental health literacy (MHL) and human rights among the new-comers, especially low-educated mothers with children. This article aimed to present a case study report of several years’ experience of tailor-made group health promotion intervention. It describes as an example, a project during autumn 2018 in five municipalities of Sweden and in collaboration between academia, primary care, social welfare, police, and an NGO. Fifty-one women, Arabic- or Somalian speaking, with 1–7 children, mean age 40 years, low-educated and in average four years’ of living in Sweden participated. Under supervision, a local female member of the NGO coordinated the group meetings in Swedish with up to ten participants and the moderators were representatives of healthcare, social services and police/lawyer, with an interpreter present. Each intervention focused on human rights, health including mental health, psychosocial and parenting support, by using a 5-week group intervention a 2 h/week, totally 10 h excluding pre- and post-evaluation, and one language per intervention. Each meeting included 1-h lecture and after a short break with refreshments, the participants asked questions to the respective moderator. It was a mixed method but emphasis on qualitative design and participatory methodology with co-creation and evaluation of the intervention. The results showed that this tailor-made group intervention gave the participants empowerment and a sense of coherence, MHL and tools to deal with stress/anxiety, based on their needs that were mapped before in a pilot study during Spring 2018, indirectly addressing their children. However, they did not primarily talk about mental illness experience. It is vital that these interventions toward the target group with limited exposure to Western concepts (e.g., illness, anxiety, and trauma), may bridge the gap between Western and traditional cultural understanding of pre- and postmigration stress. In conclusion, MHL may be a function of both the cultural origin of the target group’s background and their resettlement in a Western reception country. Implications are discussed.

## Introduction

The Lancet Commissions (1) considers that migration is a global reality but most refugees and other migrants are kept in settings where resources and skills among their needs of mental health care are lacking. During 2015–16, European countries (particularly Sweden and Germany) received the largest inflow of refugees since WWII, with over a million Syrians and others from the Middle East entering the region. As a result of a new law in Sweden, the influx then halted, “indicating the lack of preparedness of even advanced nations to deal with this humanitarian crisis” (2, page 139).

According to World Health Organization Regional Office for Europe’s first report (3), nearly 1 in 10 in the WHO European Region with 53 Member States is currently an international migrant. Further, a major reason for people to migrant besides war, civil war and persecution, natural disasters and human rights abuses are to survive by finding job. Migration and displacement are social determinants of disturbing the health of refugees and other migrants. The refugee crisis has in a quick reaction among authorities normalized a view of naïve thinking and labeling of “us” versus “them” with “ethical dimensions for people to contend with as human beings and as professionals” (4, page 18).

There is evidence that war trauma influence parenting behavior and women have a two to three times higher risk of developing post-traumatic stress disorder (PTSD) compared to men. Both psychosocial and biological reasons (oxytocin related) have been discussed (5). There is less evidence on how other forms of psychological distress (e.g., depression, anxiety, and pain) stemming from pre-and postmigration stressors as well as acculturation problems may influence parenting behavior on child psychosocial outcomes (6). A qualitative study of healthcare workers’ perceptions on parental health knowledge and child health effects among Southeast Asian American immigrants and refugees explored the following broad themes: complexity of parental health knowledge; experience from the respective home country and parent characteristics; the influence of the reception country system; help-seeking behavior from a culture point of view; and health symptoms on children (7). An interview study on the experiences of 17 Sudanese refugee women raising their teenagers in Australia, four main issues caught their main worries; such as the shift from child raising in an interdependent society as part of a network of family and community affairs (it takes a whole village to rear the child) to nurturing alone in the reception country context, mothers’ insecurity and practices of dropping their children, both literally and symbolically, defeat of parental authority, and the habits in mothers adjusted to the postmigration context and found new meaning of parenting (8). Evidence suggests that reduced mental health literacy (MHL) may be a noteworthy element influencing help-seeking behavior among refugees and other migrants with mental health problems. Yaser, Slewa-Younan, Smith, Olsson, Guajardo, and Mond (9) performed an interview study regarding problem acknowledgement and views about the benefit of activities, treatments, and treatment workers relating to a vignette as PTSD in a group of Australian based resettled Afghan refugees. It showed that approximately one-third of the participants recognized the difficulties called as PTSD but participants with less education were more in common to do it in that way, and little more than one-fourth assumed that the main problem was to be insecure. In order to overcome this problem, 18% of participants thought that to start a hobby or going out followed by better diet, improving exercise would be self-medicine. In addition, participants with less school education and older participants were more possibly to think that praying would improve their health.

In a systematic review, risk, and preventive factors related with family linked violence in refugee families show that premigration stress (trauma before arrival in a reception country) and postmigration stress factors have consequences at both an individual and a family level (10). Groen, Richters, Laban, van Busschbach, and Devillé (11) presented similar findings in a mixed-methods design study among refugees and asylum seekers in Netherlands that in addition, acculturation difficulties also contribute to misunderstanding of cultural identity. By reduction of these stress and problem factors, may elucidate cultural identity which may contribute to posttraumatic recovery.

A systematic review of supposed barriers and facilitators of mental health care utilization in adult trauma survivors shows that barriers were linked to stigma, embarrassment and rejection, low mental health competence, absence of awareness, and treatment-related doubts, distress of negative social consequences, limited resources, time, and costs (12). An epidemiological study from Santavirta, Santavirta, and Gillman (13) on the risk of mental inward among children of adults who were displaced as child refugees to Sweden from Finland during WWII support evidence regarding intergenerational associations of war-related experiences with mental health that may continue across generations. The Finnish research team noted that women of mothers who were evacuated to Sweden as a child had a raised risk for psychiatric hospitalization. Bowlby (14) described emotional attachment as a durable psychological connectedness between social beings and attachment is first created between the infant and its primary caregiver (primarily mother). If the parent is absent bodily and/or mentally, the child may develop a sense of insecurity and the age level have impact on this effect. Additionally, evidence indicates that specific cultural aspects affect the meaning and interpretation of child-rearing conditions, e.g., the meaning of the extended family in child-rearing, demanding child-rearing methods, the within-society heterogeneity, and the stability of a national level (15).

The Adaptation and Development After Persecution and Trauma (ADAPT) model has been developed by Silove (16) and recognizes five main psychosocial posts which may be disordered by conflict, separation and displacement, i.e., systems of safety and security, interpersonal bonds and networks, justice, roles and identities and existential meaning and coherence. These posts are stable in societies with peace. To perceive social exclusion as young may be a challenge to be engaged in extremist groups. Obaidi, Bergh, Akrami, and Anjum (17) reviewed studies in the topic and found that Western-born Muslims and raised in Western countries in comparison with foreign-born Muslims recorded higher on all examined judges of extremism. Daily stressors, such as social determinants (discrimination and poverty) in low resource refugee reception contexts also increase the risk of illness among the newcomers.

A systematic review showed that psychological treatment (CBT) for trauma can be effective on asylum seekers and refugees in the reception countries (18). Several international professional network organizations are in the process of evolving different solutions proposing psychosocial support to risk persons in need (19). In our increasing globalizing world, health care workers and stakeholders are more and more meeting new-coming refugees and other migrants and perceive cross-cultural challenges in which increase ethical questions such as “How to do the proper thing.” A theoretical model of more tailored solutions centered on individuals’ needs and situations can be established in collaboration with these target groups. Such a co-creation and evaluation of public health interventions show to be promising (20) but needs further study.

Torture survivors have rights to rehabilitation but the present research do not yet have a comprehensive view for the torture survivor experience (21, 22). World Health Organization Regional Office for Europe published in 2018 a technical guidance for health promotion for enhanced refugee and other migrant health that “as for all people, refugees and migrants have the fundamental right to enjoy the highest attainable standard of health….” health promotion is the process of enabling people to gain more control over, and improve, their own health and well-being, and that of their families and communities” (23, page vi). Therefore, refugee flows in the world present great challenges for how care needs should be met in the reception country. Paying attention to prevention, support, treatment, and long-term rehabilitation needs an interdisciplinary approach between healthcare, legal, and other experts (24).

For interventions to disrupt the transgenerational effect of adversity and war-related trauma, it is of significance to ask about the needs of newcomers in order to perceive security and health including mental health (20). To fill the gap in the literature, this article aimed to present a case study report of several years’ experience of tailor-made group health promotion intervention toward new-coming low-educated mothers with children. The question asked was how the participants in a tailor-made group intervention perceived their MHL before and direct after?

### The Case: Lessons learned

During the last ten years, in small-scaled projects, culturally tailored participatory health promotion group intervention toward new-coming mainly Arabic, Dari, and Somali-speaking women with war experiences have been performed. The aim has been to assess their health, with a mixed methods design before and after the group meetings with promising results and has been described in detail (25–29) and will therefore be presented briefly here. The ADAPT framework (16) and co-creation (20) have been used as a conceptual framework for formulating and implanting the intervention for new-coming mothers with low education and health literacy war experiences. The different projects due to funding have all been approved by regional ethical committees. Activity yearly grants was approved from autumn 2017 by the Public Health Agency to NGOs working with prevention mental illness among children in migration. A collaboration was set up with 1.6miljonerklubben^1^ and the author has collaborated before in an EU funding project (28).

The present project included needs-driven health literacy and human rights promotion interventions to new-coming mothers with children coming from war areas. During autumn 2018, the intervention was designed by collaboration between academy (author), primary care, social welfare, and police/lawyer and the same NGO. By supervision from the author, a local contact woman from the NGO coordinated the interventions, by inviting the women and moderators (social assistant officer, police/lawyer, midwife, psychologist, and nutrionist/physiotherapist). Prior to the intervention, focus group discussions with immigrant women who had been living in Sweden for a long period and interviews with clinicians who meet these women have been performed as a pilot study in order to gather data on the prerequisites and level of health literacy and to tailor-made the group intervention. The language was Swedish with an interpreter in the room and one language at a time. The pedagogical method was a participatory methodological approach (20) according to the topic (**Box 1**). Ten to fourteen women were called to participate in a dialog with the respective moderator. In the middle of the sitting, there was a pause for some complementary refreshments. Each group sitting comprised five occasions of 2-h per occasion, a total of 10 h excluding 1 h before and after for oral and verbal information, verbal consent and evaluation, respectively. It was closed with the reason to develop trust in the sitting. The sitting had eight basic principles. 1) The interpreter has a duty of secrecy and translates everything that is said in the room. 2) Everything that is said in the room stays in the room. 3) The mobile phone is switched off. 4) The concentration on the here and now and feeling mentally good. 5) Religion and politics as well as economy and housing can be discussed in other contexts. 6) For questions and comments, the hand is raised. 7) It’s a closed group—no one comes and goes. 8) No information is given to authorities. (Privacy is the main rule, but there are several exceptions such as reporting and reporting obligations by Swedish law for the moderators).

Box 1 **Description of intervention by themes with different moderators working locally (can be in different order locally)**

The intervention comprises a 2-h sitting/week for 5 weeks, totally 10 h excluding 1-h information and consent and 1-h evaluation on the following topics:

Theme 1. Women and human rights issue (police/lawyer)
Theme 2. Children and family (social assistant officer)
Theme 3. Women’s body, health and health care organization (midwife)
Theme 4. Mental health, stress and recovery (licensed psychologist)
Theme 5. Health promotion, e.g., diet, exercise and wellness (nutrionist/physiotherapist)

It was found in the pilot study in Spring 2018 that women with low or no education were unable to answer the five questions in EQ-5D^2^ but to keep the Likert scale of perceived health, 0–100. The central question before and after the group meetings was not mental health/illness and suicide prevention, but about anxiety/stress with three response options (none, to a certain extent and to a great extent) with subsequent questions with open response options. Before: If you feel anxiety/stress, what do you feel is the cause of your anxiety/stress? If you feel anxiety/stress, what do you usually do to feel better? After: If you feel anxiety/stress, what do you experience after the group intervention are the cause of your anxiety/stress? What knowledge and tools have you gained during the group meetings to feel less anxiety/stress and feel better?

#### Main Concern Was That They Felt Stress and Fear About the Future

Fifty-one women, Arabic- or Somalian speaking, with 1–7 children, mean age 40 years, low- or no education from home country and in average four years’ of living in Sweden participated during autumn 2018. Fifty-six percent of women replied that before the group intervention they experienced to some extent or to a great extent anxiety/stress and afterward the figure was 42%. On the Likert scale (0–100), the women felt that they averaged on perceived health 52 before the group intervention, while the figure after had been increased to an average of 68, which was a significant improvement (p < .001). They got useful tools to deal with stress/anxiety based on their needs that were mapped before. For instance, before the group intervention, they felt insecure and stress about future for their children and rest of the family. They were interested to know about the children’s activities at school. Illustrations of questions from participants related to different themes and were like an earlier study (**Table 1**). After the intervention they felt more comfortable to talk with their children, and they learnt that they can say “no” to the child. Before the intervention, they were anxious that the social welfare would take the child because a bruise which they thought would be interpreted as they beat the child which is forbidden according to law in Sweden.

**Table 1:** Illustrations of questions from participants related to the different themes (27).

Theme	Question
Swedish health-care system	If I do not speak Swedish well enough, how do I go about calling the healthcare center for an appointment?
Health eating habits	Can I develop cancer from not eating healthy?
Dental care	Can my gums disappear if they are bleeding while I brush my teeth?
Physical activity	How can the physiotherapist help to ease my pain?
Stress	Can mental illness affect my memory?
Female anatomy	If I am only allowed to wash my vagina once a day, how do I go about it when I need to pray? I must be clean then!
Social service support for parents and children	When does the social service place children in foster care?
Domestic violence	For how long a person is detained for domestic violence?

### Implications

The experience of these tailor-made interventions highlights two main findings: the first finding was that women felt empowered by receiving facts from police, social assistant officer, and health care workers and the intervention inspired them to change their lifestyle to healthier one for the whole family including the children and increased the trust to authorities. Gender-separate groups may enable facts uptake, while dialogue regarding sexual health norms may have advantage from performing in assorted groups (30). They became ambassadors of a preventive perspective as training their children at a primary age about healthy living behaviors increases the odds of keeping these customs in grownups life which may have a constructive influence on public health in general. The second finding was that the women expressed a wish to continue the learning process, were more interested in learning Swedish, and confident of take care of their own health. It was not clear for them before the intervention that they own their own body. A study showed that a substantial amount of the refugees in Sweden have incomplete comprehensive health literacy and account less than good health and impaired well-being, or that they have avoided doing from seeking health care (31). Therefore, such preventive intervention is of significance which may also increase social inclusion and integration.

The UN Agenda for Sustainable Development 2030 (32) offers a global plan for dignity, peace and wealth for people and the planet, now and in the future. There is a risk that the goals will not be reached if the contemporary refugee crisis persist and increase. Priebe, Sandu, Dias, Gaddini, Greacen, Ioannidis et al.’s (33) study of good practice closed that “Service delivery should be achieved by dispensing care on an individual basis, considering personal need, rather than focusing on group stereotypes and customary nations that migrants need may differ greatly from indigenous patients” (page 196). In sum:

- Systematically identify the new-coming women’s situation, needs, risk factors
- Increase awareness of health care providers regarding and how they can affect
- Address socioeconomic barriers such as social determinants of health (poor living conditions, low educated and unemployment, as well as associated stress, to reduce negative impacts on their children and other family members)
- Promote improvement of health literacy levels, engaging all relevant stakeholders, before the new-coming women are identified as patients
- Promote social inclusion and integration.

### Conclusion

MHL, culturally tailor-made group interventions would pay attention to human rights, women’s health, psychosocial and childrearing for war-affected parents, especially mothers with low or no education, by using a participatory methodology, such as co-creation and evaluation of public health interventions (20). Further, it is vital that these interventions toward newcomers with low MHL and with limited exposure to Western concepts (e.g., illness, anxiety, and trauma), may bridge the gap between Western and traditional cultural understanding of pre- and postmigration stress, which addressing indirectly the children.

It is also time to reflect on our current reception paradigms in meeting the needs of new-coming refugees and other migrants and how we may react to a worsening worldwide emergency for which present methods in the literature are not enough to reach the large numbers of new-comers in need. A “smörgåsbord” of comprehensive health promotion and intervention approaches with interpersonal and intercultural training of staff may facilitate social inclusion and integration of the target group.

## Data Availability Statement

The original contributions presented in the study are included in the article/supplementary material, further inquiries can be directed to the corresponding author.

## Ethics Statement

The studies involving human participants were reviewed and approved by Regionala etikprövningsnämnden Stockholm 2018/224-31/5. The participants provided their written informed consent to participate in this study.

## Author Contributions

The author confirms being the sole contributor of this work and has approved it for publication.

## Funding

This work was supported by the Public Health Agency of Sweden (03459-2017-6.2).

## Conflict of Interest

The author declares that the research was conducted in the absence of any commercial or financial relationships that could be construed as a potential conflict of interest.

## Footnotes

1
www.1.6miljonerklubben.com

2
http://euroqol.org

## References

[1] The Lancet Commissions The UCL-Lancet Commission on Migration and Health: the health of a world on the move. www.thelancet.com, online December 5, 2018. (2018) 392(10164):2606–54. 10.1016/S0140-6736(18)32114-7. PMC7612863 30528486

[2] Silove D Ventevogel P and Rees S The contemporary refugee crisis: an overview of mental health challenges. World Psychiatry (2017) 16:130–9. 10.1002/wps.20438 PMC5428192 28498581

[3] World Health Organization Report on the health of refugees and migrants in the WHO European Region: no public health without refugee and migrant health. Copenhagen, Denmark: Regional Office for Europe (Copenhagen): WHO (2018). http://www.euro.who.int/en/publications/abstracts/report-on-the-health-of-refugees-and-migrants-in-the-who-european-region-no-public-health-without-refugee-and-migrant-health-2018.

[4] Krausz RM Choice F Psychiatry"s response to mass traumatisation and the global refugee crisis. Lancet Psychiatry (2017) 4(1):18–20. 10.1016/S2215-0366(16)30413-8 28012471

[5] Christiansen DM Hansen M Accounting for sex differences in PTSD: a multi-variable mediation model. Eur J Psychotraumatol (2015) 6:26068. 10.3402/ejpt.v6.26068. 25604705 PMC4300366

[6] Sim A Bowes L Garner F Modeling the effects of war exposure and daily stressors on maternal mental health, parenting and child psychosocial adjustment; a cross-sectional study with Syrian refugees in Lebanon. Global Ment Health (2018) 5:1–12:e40. 10.1017/gmh.2018.33 PMC6315283 30637113

[7] Khuu BP Lee HY Zhou AQ Shin J Lee RM Healthcare providers’ perspectives on parental health literacy and child health outcomes among Southeast Asian American immigrants and refugees. Children Youth Serv Rev (2016) 67:220–9. 10.1016/j.childyouth.2016.06.006

[8] Levi M Mothering in transition: The experiences of Sudanese refugee women raising teenagers in Australia. Transcult Psychiatry (2014) 51:479. 10.1177/1363461514531315 24803540

[9] Yaser A Slewa-Younan S Smith CA Olson RE Guajardo MGU Mond J Beliefs and knowledge about post-traumatic stress disorder amongst resettled Afghan refugees in Australia. Int J Ment Health Syst (2016) 10:31. 10.1186/s13033-016-0065-7 27073412 PMC4828823

[10] Timshel I Montgomery E Thorup Dalgaard N A systematic review of risk and protective factors associated with family related violence in refugee families. Child Abuse Negl (2017) 70:315–30. 10.1016/j.chiabu.2017.06.023 28683372

[11] Groen SPN Richters AJM Laban CJ van Busschbach JT Devillé WLJM Cultural identity confusion and psychopathology. A mixed-methods study among refugees and asylum seekers in the Netherlands. J Nerv Ment Dis (2019) 207(3):162–70. 10.1097/NMD.0000000000000935 PMC6392308 30724831

[12] Kantor V Knefel M Lueger-Schuster B Perceived barriers and facilitators of mental health service utilization in adult trauma survivors: a systematic review. Clin Psychol Rev (2017) 52:52–68. 10.1016/j.cpr.2016.12.001 28013081

[13] Santavirta T Santavirta N Gillman SE Association of the World War II Finnish evacuation with psychiatric hospitalization in the next generation of children. JAMA Psychiatry (2017) 75(1):21–7. 10.1001/jamapsychiatry.2017.3511 PMC5833542 29188292

[14] Bowlby J Attachment and loss. Loss, sadness and depression. J Biosoc Sci (1981) 13(03):1–355. 10.1017/S0021932000013596 7462265

[15] Zevulun D Kalverboer ME Zijlstra AE Post WJ Knorth EJ Returned migrant children in Kosovo and Albania: assessing the quality of child-rearing from a non-western perspective. Cross-Cult Res (2015) 49(5):489–521. 10.1177/1069397115608173

[16] Silove D The psychosocial effects of torture, mass human rights violations, and refugee trauma: toward an integrated conceptual framework. J Nerv Ment Dis (1999) 187(4):200–7. 10.1097/00005053-199904000-00002 10221552

[17] Obaidi M Bergh R Akrami N Anjum G Group-based relative deprivation explains endorsement of extremism among Western-born Muslims. Psychol Sci (2019) 30(4):596–605. 10.1177/0956797619834879 30875267

[18] Nose M Ballette F Boghelli I Turrini G Purgato M Tol W Psychosocial interventions for post-traumatic stress disorder in refugees and asylum seekers resettled in high-income countries: a systematic review and meta-analyis. PloS One (2017) 12(2):e0171030. 10.1371/journal.pone.0171030 28151992 PMC5289495

[19] Murray KE Davidson GR Schweitzer RD Review of refugee mental health interventions following resettlement: best practices and recommendations. Am J Orthopsychiat (2010) 80:576–85. 10.1111/j.1939-0025.2010.01062.x PMC3727171 20950298

[20] Leask CF Sandlund M Skelton DA Altenburg TM Cardon G Chinapaw MJM Framework, principles and recommendations for utilising participatory methodologies in the co-creation and evaluation of public health interventions. Res Involvement Engagem (2019) 5:2. 10.1186/s40900-018-0136-9 PMC6327557 30652027

[21] Sjölund BH Kastrup M Montgomery E Persson AL Rehabilitating torture survivors. J Rehabil Med (2009) 41:689–96. 10.2340/16501977-0426 19774300

[22] Liddell BJ Nickerson A Bryant RA Clinical science and torture survivors" rights to rehabilitation. Lancet Psychiatry (2018) 5(2):P101–3. 10.1016/S2215-0366(17)30332-2 29102313

[23] World Health Organization Health promotion for improved refugee and migrant health. Technical guidance. Regional Office for Europe (Copenhagen): WHO (2018). http://www.euro.who.int/:data/assets/pdf_file/0004/388363/tc-health-promotion-eng.pdf?ua=1.

[24] Wenzel TH Ekblad S Kastrup M Musisi S Torture and sequels to persecution – a global challenge. In: Javed A. Fountoulakis KN, editors. Advances in Psychiatry Chan, Switzerland: Springer International Publishing AG and World Psychiatric Association (2018) p. 405–24. 10.1007/978-3-319-70554-5_25

[25] Ekblad S Behovsstyrda cirkelträffar i grupp för nyanlända kvinnor med barn minskar upplevd oro/stress om framtiden. En kvalitetssäkring på fem orter (Need-driven group meetings for newly arrived women with children reduce experienced worries / stress about the future. A quality assurance in five locations). Social Medicinsk Tidskr (2019) 96(1):85–94.

[26] Ekblad S Asplund M Culture- and evidence-based health promotion group education perceived by new-coming adult Arabic-speaking male and female refugees to Sweden - pre and two post assessments. Open J Prevent Med (2013) 3:12–21. 10.4236/ojpm.2013.31002

[27] Ekblad S Hjerpe A Lunding H Tailor-made group training by clinical staff to empower equity in health towards newly arriving Arabic- and Somali speaking women in Sweden. Public Health Panor: Topic Migr Health Eur Region J WHO Reg Off Eur (2016) 2(4):466–76.

[28] Ekblad S Persson-Valenzuela U-B Lifestyle course is an investment in perceived improved health among newly arrived women from countries outside Europe. Int J Environ Res Public Health (2014) 11:10622–40. 10.3390/ijerph111010622 PMC4210998 25321877

[29] Eriksson-Sjöö T Cederberg M Östman M Ekblad S Quality of life and health promotion intervention – a follow up study among Newly-arrived Arabic Speaking Refugees in Malmö, Sweden. Int J Migr Health Soc Care (2012) 8:112–26. 10.1108/17479891211267302

[30] Svensson P Carlzén K Agardh A Exposure to culturally sensitive sexual health information and impact on health literacy: a qualitative study among newly arrived refugee women in Sweden. Cult Health Sex (2017) 19(7):752–66. 10.1080/13691058.2016.1259503. 27894219

[31] Wångdahl J Lytsy P Mårtensson L Westerling R Poor health and refraining from seeking healthcare are associated with comprehensive health literacy among refugees: a Swedish cross-sectional study. Int J Public Health (2018) 63::409–19. 10.1007/s00038-017-1074-2 PMC5978924 29480325

[32] The United Nations The Sustainable Development Goals Report. New York: The United Nations (2018). https://unstats.un.org/sdgs/files/report/2018/TheSustainableDevelopmentGoalsReport2018-EN.pdf

[33] Priebe S Sandu S Dias S Gaddini A Greacen T Ioannidis E Good practice in health care for migrants: views and experiences of care professionals in 16 European countries. BMC Public Health (2011) 11::1987. 10.1186/1471-2458-11-187 PMC3071322 21439059

